# Examining provider practice-level disparities in delivery outcomes among patients with a history of Cesarean Delivery

**DOI:** 10.1186/s12884-024-06458-3

**Published:** 2024-04-05

**Authors:** Lily McCarthy, Nicola F Tavella, Sara Wetzler, Lily Ardente, Molly Chadwick, Dexter Paul, Nikki Sabet, Toni Stern, Angela Bianco

**Affiliations:** 1 Graduate School of Biomedical Sciences, Icahn School of Medicine at Mount Sinai Hospital, New York, NY 10029 USA; 2 Department of Obstetrics, Gynecology, and Reproductive Science, Icahn School of Medicine at Mount Sinai Hospital, New York, NY 10029 USA; 3 Princeton University, Princeton, NJ 08544 USA; 4 Maternal-Fetal Medicine, Icahn School of Medicine at Mount Sinai Hospital, New York, NY 10029 USA

**Keywords:** Obstetrics, Pregnancy, Trial of labor, Midwifery, Retrospective studies, Multivariate analysis

## Abstract

**Background:**

Choosing whether to pursue a trial of labor after cesarean (TOLAC) or scheduled repeat cesarean delivery (SRCD) requires prenatal assessment of risks and benefits. Providers and patients play a central role in this process. However, the influence of provider-associated characteristics on delivery methods remains unclear. We hypothesized that different provider practice groups have different obstetric outcomes in patients with one prior cesarean delivery (CD).

**Methods:**

This was a retrospective cohort study of deliveries between April 29, 2015 – April 29, 2020. Subjects were divided into three cohorts: SRCD, successful VBAC, and unsuccessful VBAC (patients who chose TOLAC but had a CD). Disparities were reviewed between five different obstetric provider practice groups, determined from a breakdown of different providers delivering at the study site during the study period. Proportional differences were examined using Chi-squared tests and logistic regression models.

**Results:**

1,439 deliveries were included in the study. There were significant proportional disparities between patients in the different groups. Specifically, patients from Group D were significantly more likely to undergo successful VBAC, while patients seeing a provider from Group A were more likely to deliver by SRCD. In our multivariate analysis of successful versus unsuccessful VBAC, patients from Group D had greater odds ratios of successful VBAC compared to Group A. Patients delivered by Group E had a significantly lower odds ratio of successful VBAC.

**Conclusion:**

This study suggests an association between provider practice groups and delivery outcomes among patients with one prior CD. These data contribute to a growing body of literature around patient choice in pregnancy and the interplay of patients and providers. These findings help to guide future investigations to improve outcomes among patients with a history of CD.

## Background

Cesarean deliveries (CDs), including scheduled repeat cesarean deliveries (SRCDs), have become more common [1, 2]. However, a trial of labor after cesarean (TOLAC) is safe for most women [3]. Successful vaginal birth after cesarean (VBAC) is associated with lower maternal mortality and morbidity, faster recovery, and decreased future complications [4–6]. Nevertheless, morbidity outcomes from unsuccessful VBAC may be worse than those of a SRCD, particularly in the case of uterine rupture [7]. One recent study found uterine rupture during TOLAC associated with a host of adverse maternal and neonatal health outcomes [8]. Thus, the choice of delivery method involves risk-benefit analyses during shared decision-making discussions between patients and providers.

There is emerging interest in how provider differences may influence decisions about delivery mode [9–11]. Recent research reaffirms that patients often defer to their providers regarding delivery [12]. TOLAC preference appears more common among midwives, while SRCD is more prevalent among obstetricians (OBs) [13–16]. TOLAC also seems more prevalent among laborists (attending physicians employed by the institution to staff the labor floor and tend to any patient in labor) [17]. Other factors related to provider characteristics including differences in call schedule and affective traits have also been analyzed [10, 18]. Differences in preferences between providers and patients have been reported, with qualitative influences on delivery [19].

Nevertheless, there are still lingering questions about provider practice-related dynamics. Little is known about the potential effects of differences in patient preferences and provider practice settings (including private general practices and maternal-fetal medicine practices). One study evaluated the significant implications of patient cultural factors on delivery mode preference [20]. Another study examined factors associated with patient demand for CD, with a history of infertility increasing likelihood of CD [21]. In the current study, our primary goal was to determine whether differences in delivering provider practice correlated with higher likelihood of TOLAC or SRCD. We hypothesized that patients with a history of cesarean delivered by different provider practice groups would experience different rates of outcomes related to mode of delivery and associated demographic and clinical characteristics.

## Methods

This was a retrospective cohort study of patients with a history of CD who delivered at a single major urban hospital in New York City between April 29, 2015 – April 29, 2020. For reference, in 2022 this institution conducted 6,956 deliveries, of which 27.8% were by CD. We serve a large, diverse population of patients with varying levels of social vulnerability. The Icahn School of Medicine at the Mount Sinai Hospital Institutional Review Board (IRB) oversaw the conduct of this project. Chart review abstracted demographic and clinical data, including race and ethnicity, health insurance, pre-pregnancy body mass index (BMI), third-trimester sonographic estimated fetal weight (EFW), census-tract specific Social Vulnerability Index (SVI), and provider-level information related to the delivering clinician. SVI values were dichotomized as “low-medium” if the index value was < 0.75 and “high” if the value was $\ge$ 0.75. We excluded patients with multiple gestations and a history of more than one CD, as well as patients who delivered by cesarean for contraindications of TOLAC (malpresentation, placenta previa, vasa previa, history of myomectomy, suspected placenta accreta spectrum, fetal anomalies, and unplanned clinical indications including hypertensive disorders, cardiac abnormalities, and active bleeding). Patients were grouped based on whether they opted for TOLAC and delivered by VBAC or CD. This yielded three groups: successful VBAC, unsuccessful VBAC, and SRCD. We divided the study sample in this way to examine odds of successful versus unsuccessful VBAC among different provider practice groups.

The five categories of delivering provider practices included Group A, private general obstetrician groups of varying sizes; Group B, a large non-profit OB practice serving a predominately publicly insured population with a strong cultural proclivity for vaginal birth; Group C, private/academic maternal-fetal medicine (MFM) providers; Group D, midwifery providers serving patients mostly with public insurance; and Group E, the laborist-trainee group that also primarily serves patients with public insurance. Counseling regarding mode of delivery occurs during prenatal care by the same providers who ultimately deliver the patient. Our institutional standard of care requires patients with a single prior CD to sign a TOLAC consent form prenatally that must be re-attested at the time of admission for delivery.

Disparities between different obstetric provider practice groups were examined, with delivery outcome as the basis for analysis. Proportional differences of demographic and clinical characteristics were analyzed between different provider practice groups using Chi-squared tests. A two-sided *p* < 0.05 was considered statistically significant. We examined the likelihood of successful versus unsuccessful VBAC using a pair of logistic regression models, with the multivariate model controlling for age, pre-pregnancy BMI, gestational age at delivery (in weeks), birthweight (in grams), history of prior vaginal delivery and VBAC, and indications for the primary CD. The statistical program R housed these analyses [22].

## Results

1,439 patients delivered a pregnancy after a previous cesarean during the study interval who met inclusion criteria. 993 patients had a successful VBAC (69%) while 205 patients had a SRCD (14%) and 241 had an unsuccessful VBAC resulting in CD (17%).

Table 1 displays demographic characteristics of the sample by provider practice type.

**Table 1:** Demographic Characteristics

	Group A		Group B		Group C		Group D		Group E			
	Total	474	Total	299	Total	313	Total	61	Total	292		
**Characteristic**	N	%	N	%	N	%	N	%	N	%	χ2	*p*
Age at Index Delivery (years)											213	< 0.001
< 25	6	1.3	25	8.4	11	3.5	6	9.8	23	7.9		
25–30	49	10.3	108	36.1	64	20.4	20	32.8	114	39.0		
31–35	176	37.1	72	24.1	99	31.6	25	41.0	104	35.6		
36–40	201	42.4	60	20.1	98	31.3	8	13.1	44	15.1		
> 40	42	8.9	34	11.4	41	13.1	2	3.3	7	2.4		
Gestational Age at Index Delivery (weeks)											68	< 0.001
< 28	2	0.4	0	0.0	3	1.0	0	0.0	2	0.7		
29–36	46	9.7	33	11.0	77	24.6	6	9.8	50	17.1		
37–40	391	82.5	216	72.2	217	69.3	47	77.0	209	71.6		
> 40	35	7.4	50	16.7	16	5.1	8	13.1	31	10.6		
Social Vulnerability Index (SVI)											354	< 0.001
Low-Medium	350	73.8	244	81.6	223	71.2	9	14.8	61	20.9		
High	124	26.2	55	18.4	90	28.8	52	85.2	231	79.1		
Race & Ethnicity											973	< 0.001
White	330	69.6	297	99.3	261	83.4	6	9.8	14	4.8		
Asian	39	8.2	1	0.3	13	4.2	3	4.9	14	4.8		
Black/African American	28	5.9	1	0.3	7	2.2	10	16.4	127	43.5		
Hispanic/Latina	38	8.0	0	0.0	13	4.2	37	60.7	129	44.2		
Other	39	8.2	0	0.0	19	6.1	5	8.2	8	2.7		
Health Insurance											960	< 0.001
Private	419	88.4	24	8.0	273	87.2	4	6.6	32	11.0		
Public	33	7.0	267	89.3	24	7.7	55	90.2	253	86.6		
None	22	4.6	8	2.7	16	5.1	2	3.3	7	2.4		
Pre-pregnancy BMI											174	< 0.001
< 30	317	66.9	162	54.2	201	64.2	24	39.3	90	30.8		
30–34.9	83	17.5	90	30.1	70	22.4	25	41.0	100	34.2		
35–39.9	24	5.1	25	8.4	19	6.1	4	6.6	51	17.5		
>/= 40	9	1.9	13	4.3	8	2.6	6	9.8	41	14.0		
Missing	41	8.6	9	3.0	15	4.8	2	3.3	10	3.4		
Third-trimester EFW											21	< 0.01
< 25th Percentile	12	2.5	1	0.3	11	3.5	1	1.6	10	3.4		
25-75th Percentile	194	40.9	8	2.7	58	18.5	23	37.7	165	56.5		
75th Percentile	56	11.8	0	0.0	14	4.5	8	13.1	40	13.7		
Missing	212	44.7	290	97.0	230	73.5	29	47.5	77	26.4		

Age at delivery was significantly different between provider practice groups; 13% of patients in Group C were older than 40 years, compared to 2% of patients delivered by Group E (*p* < 0.001). Differences were noted by gestational age at delivery; 26% of Group C patients delivered before 37 weeks’ gestation compared to 10% of patients delivered by Group A. Conversely, 17% of patients from Group B delivered at > 40 weeks’ gestation, compared to 5% of Group C patients (*p* < 0.001). Different provider groups served patients at different levels of social vulnerability; 79% of patients delivered by Group E lived in high-vulnerability areas compared to 26% of patients delivered by Group A (*p* < 0.001). Provider practice groups differed significantly by race and ethnicity; 99% of patients in Group B identified as White compared to just 5% of patients delivered by Group E (*p* < 0.001). Patients in different practice groups had significantly different rates of private and public insurance; 74% of patients delivered by Group A had private insurance, compared to just 15% of patients delivered by Group D (*p* < 0.001). Pre-pregnancy BMI also differed significantly—14% of patients delivered by Group E had a pre-pregnancy BMI of Class III Obesity compared to 2% of patients delivered by Group A (*p* < 0.001). A significant proportion of subjects in all provider practice groups were missing third-trimester EFW data; for the available data, there were significant differences between provider practice groups (*p* < 0.01).

Table 2 shows the distribution of clinical characteristics by provider practice group.

**Table 2:** Indications for Primary Cesarean

	Group A		Group B		Group C		Group D		Group E			
	Total	474	Total	299	Total	313	Total	61	Total	292		
**Characteristic**	N	%	N	%	N	%	N	%	N	%	χ2	*p*
Median Gravidity (IQR)	3 (2)		6 (6)		4 (4)		3 (2)		4 (3)			
Median Term Parity (IQR)	1 (0)		4 (4)		1 (2)		1 (1)		1 (1)			
History of Vaginal Delivery											217	< 0.001
Yes	78	16.5	196	65.6	104	33.2	25	41.0	70	24.0		
No	396	83.5	103	34.4	209	66.8	36	59.0	222	76.0		
History of VBAC											181	< 0.001
Yes	66	13.9	171	57.2	102	32.6	8	13.1	68	23.3		
No	408	86.1	128	42.8	211	67.4	53	86.9	224	76.7		
Indication for 1’CD: Malpresentation											37	< 0.001
Yes	108	22.8	94	31.4	85	27.2	5	8.2	40	13.7		
No	366	77.2	205	68.6	228	72.8	56	91.8	252	86.3		
Indication for 1’CD: Elective											10	0.04
Yes	16	3.4	1	0.3	6	1.9	2	3.3	4	1.4		
No	458	96.6	298	99.7	307	98.1	59	96.7	288	98.6		
Indication for 1’CD: Multiple Gestation											33	< 0.001
Yes	10	2.1	23	7.7	29	9.3	0	0.0	7	2.4		
No	464	97.9	276	92.3	284	90.7	61	100.0	285	97.6		
Indication for 1’CD: Failed Induction of Labor											9	0.05
Yes	29	6.1	14	4.7	15	4.8	5	8.2	29	9.9		
No	445	93.9	285	95.3	298	95.2	56	91.8	263	90.1		

Median gravidity was highest for Group B patients (6, IQR:6) and lowest for Groups A and D patients (3, IQR:2). Median term parity was highest for Group B patients (4, IQR:4) and lowest for Group A patients (1, IQR:0). Patient groups differed significantly by vaginal delivery history; 66% of Group B patients had a previous vaginal delivery compared to 17% of Group A patients (*p* < 0.001). 57% of Group B patients had a VBAC before the delivery indexed in the present study, compared to 14% of Group A patients (*p* < 0.001). There were also significant proportional differences with several primary cesarean indications as displayed in Table 2.

Table 3 displays proportional differences in delivery outcomes among different provider practice groups.

**Table 3:** Delivery Outcomes

	Group A		Group B		Group C		Group D		Group E			
	Total	474	Total	299	Total	313	Total	61	Total	292		
**Characteristic**	N	%	N	%	N	%	N	%	N	%	χ2	*p*
Mode of Delivery											224	< 0.001
SRCD	136	28.69	4	1.34	45	14.38	0	0.00	20	6.85		
Successful VBAC	252	53.16	273	91.30	221	70.61	60	98.36	187	64.04		
Unsuccessful VBAC	86	18.14	22	7.36	47	15.02	1	1.64	85	29.11		
Hysterectomy during Index Delivery											2	0.6
Yes	2	0.4	2	0.7	4	1.3	0	0.0	2	0.7		
No	472	99.6	297	99.3	309	98.7	61	100.0	290	99.3		
Uterine Rupture during Index Delivery											0.3	1
Yes	1	0.2	1	0.3	1	0.3	0	0.0	1	0.3		
No	473	99.8	298	99.7	312	99.7	61	100.0	291	99.7		
Placental Abruption during Index Delivery											1	0.8
Yes	4	0.8	4	1.3	3	1.0	0	0.0	4	1.4		
No	470	99.2	295	98.7	310	99.0	61	100.0	288	98.6		
NICU Admission											22	< 0.001
Yes	29	6.1	14	4.7	37	9.0	9	14.8	36	12.3		
No	445	93.9	285	95.3	376	91.0	52	85.2	256	87.7		

There were significant proportional differences regarding mode of delivery (*p* < 0.001). 91% of patients delivered by Group B providers had a successful VBAC, compared to 53% of patients delivered by Group A. 29% of Group A patients delivered by SRCD, compared to zero patients delivered by Group D. 29% of patients delivered by Group E had an unsuccessful VBAC, compared to 1% of patients delivered by Group D. Rates of severe intrapartum complications (uterine rupture, hysterectomy, placental abruption) did not differ significantly between different provider practice groups. Neonates of subjects delivered by different provider practice groups differed significantly by admission to the Neonatal Intensive Care Unit (NICU), with a greater proportion of neonates from the Group D (15%) going to the NICU (*p* < 0.01). There were not adequate data on other neonatal outcomes for reporting in this paper.

Table 4 displays the results of two logistic models, one univariate and one multivariate regression, predicting odds ratios of successful VBAC versus unsuccessful VBAC.

**Table 4:** Logistic regression models predicting mode of delivery

	Predicting odds ratio of successful VBAC vs. unsuccessful VBAC			
	Univariate		Multivariate*	
	OR	95% CI	AOR	95% CI
**Delivering Provider Practice Type**				
Group A (REF)				
Group B	4.2	2.6, 7.1	1.1	0.6, 2.1
Group C	1.6	1.1, 2.4	0.8	0.5, 1.3
Group D	20.5	4.4, 364.6	15.4	3.2, 27.7
Group E	0.8	0.5, 1.1	0.6	0.4, 0.8

*Multivariate regressions adjusted for age, social demographics, pre-pregnancy BMI, gestational age at delivery (in weeks), third trimester estimated fetal weight via ultrasound, history of prior vaginal delivery, and indications for first cesarean delivery

The multivariate model adjusted for previously identified covariates. In the multivariate model, patients in Group D had a significantly greater odds ratio of successful VBAC compared to Group A (15.4; 95% CI: 3.2, 27.7). Patients delivered by Group E, by contrast, had a significantly lower odds ratio of successful VBAC (0.6, 95% CI: 0.4, 0.8).

## Discussion

This study reveals provider practice differences in delivery outcomes. While patients of Group B frequently chose TOLAC and delivered by VBAC, patients of Group A frequently chose SRCD. In multivariate models, patients delivered by Group D had significantly greater odds of successful VBAC versus unsuccessful VBAC compared to Group A. Patients delivered by Group E had significantly lower odds of successful VBAC compared to patients delivered by Group A.

Repeat CD was more prevalent in patients of private OBs, which is consistent prior data [14, 16]. Rosenstein et al. compared VBAC rates between a private practice and a collaborative midwifery-laborist model and reported decreased VBAC among private OBs. With a combined midwifery-laborist system, the VBAC rate rose by 11% [15]. Metz et al. similarly found that patients of family practitioners more often selected TOLAC [12]. It should be noted that, at our institution, midwives do not perform CDs and their patients who require such intervention are delivered by the Laborist group. This therefore misrepresents the true rate of SRCD among patients in the midwifery group.

Importantly, different provider practice groups care for significantly different patient populations. These populations differ by demographic and clinical characteristics, as Tables 1 and 2 show. MFM specialists, for example, care for high-risk pregnancies, while midwives serve a lower risk population. For example, more than twice as many patients delivered preterm in the MFM group, likely iatrogenic in many cases. As revealed in this study, private OB patients are overwhelmingly insured by private health plans. Further, certain patient groups live in areas characterized by high social vulnerability, and this impacts prenatal care [23]. Finally, patients of different practices differed significantly across indications for their primary CD.

Prior studies have suggested associations between midwifery and TOLAC. Patients of midwives represent a self-selected population with preferences for vaginal birth [12, 13]. The increased prevalence of TOLAC may reflect the philosophy of midwifery: women have the natural capacity to give birth without routine intervention [23–25]. A multicenter study found midwives were less likely to feel planned birth indications as “necessary” compared to other delivering providers [26]. Among both OBs and midwives, fear of malpractice litigation has clinical practice; one US survey found an incidence of litigation among midwives at 32%, while another indicated correlation between fear of litigation and CD rates [27–29]. Fear of litigation can influence provider choices regarding provision of care for their patients.

We found that TOLAC and VBAC were more common among certain provider groups, like Group B. This group comprises an OB practice that serves a patient population with strong cultural drivers for birth proliferation as well as avoidance of CD. We included this group to gain insight into cultural determinants of the delivery decision-making process. Only Pomeranz et al. have examined the relationship between patient-level cultural factors and delivery approach. Nearly one-third of their participants reported that providers had the strongest impact on their delivery method decision [20]. Further prospective investigation stands to illuminate these decision-making pathways [30].

Strengths of this study include the large, diverse cohort and minimal loss to follow-up. Overall, our sample was heterogeneous and included patients with varying probability of VBAC success. Some patients with one or multiple prior vaginal deliveries may have had greater odds of TOLAC and successful VBAC. Nevertheless, that was not our major area of focus as we were more concerned with provider-practice level differences and the overall process of decision making about the delivery approach.

Limitations include the fact that the study was retrospective and was based at a single institution, limiting generalizability. Examining different provider practice groups introduces complexity in that different providers provide slightly different care; this is apparent in the missing data for third-trimester EFW, where different provider practice groups varied significantly in the proportion of their patients that received a recorded third-trimester EFW ultrasound. Statistically, this study was limited in that some patient populations were ethnically homogenous or too small for sufficient comparison. This can be seen in the rates of VBAC among Groups B and D, exceeding 90%, which outpaces that reported and literature and is likely attributable to the homogeneous cultural drivers towards pursuit of VBAC in these patient populations. Another limitation relates to operationalizing provider practice type, determined based on authorship of the labor and delivery note in patients’ electronic medical records. Categorizing providers this way may not reflect which provider counseled patients. We cannot disentangle associations related to provider-level factors and patient-level factors, as patients may choose their providers, and in turn, their mode of delivery. Patients have personal preferences, and one can assume patients choose providers that align with those preferences when possible. There is therefore opportunity for selection bias. This is particularly true for Groups B and D. There is ample literature supporting the preference of midwife patients for TOLAC [12, 13, 23–25]. Thus, it is certainly possible that patients drove certain delivery patterns. Additionally, while midwives are equally involved in prenatal counseling, they are less involved in surgical practice, which introduces bias. There were limitations regarding sample size and distribution such that certain confidence intervals were imprecise in the multivariate models; a larger sample size would resolve this. Finally, we chose to group patients who received care from laborists and residents under one category (Group E), but there are likely differences between these providers.

## Conclusions

Our results suggest significant disparities between different provider practice groups regarding mode of delivery for patients with a history of CD, in ways that appreciably affect VBAC success. These data also illuminate under-researched social drivers of health that influence maternal delivery outcomes. The findings from the present study contribute to ways in which the patient-provider interaction influences outcomes in obstetrics.

## Data Availability

Health data included in this study were abstracted from medical records electronically housed in our medical institution. Limited data sets are available by request to the corresponding author as allowed by institutionally governed data transfer agreements.

## Abbreviations

CD: Cesarean delivery
OB: Obstetrician
TOLAC: Trial of labor after cesarean
VBAC: Vaginal birth after cesarean
SRCD: Scheduled repeat cesarean delivery
MFM: Maternal–fetal medicine
HIPAA: Health Insurance Portability and Accountability Act
IRB: Institutional review board
PPHS: Program for the protection of human subjects
BMI: Body mass index
